# Developing Stories From the Field to Highlight Policy, Systems, and Environmental Approaches in Obesity Prevention

**DOI:** 10.5888/pcd10.120141

**Published:** 2013-02-14

**Authors:** Marissa Zwald, Jan Jernigan, Gayle Payne, Rosanne Farris

**Affiliations:** Author Affiliations: Jan Jernigan, Gayle Payne, Rosanne Farris, Centers for Disease Control and Prevention, Atlanta, Georgia.

## Abstract

As obesity prevention initiatives increasingly shift toward approaches focused on policy, systems, and environmental change, opportunities to share experiences from the field and lessons learned are growing. Stories are a tool to illustrate processes and outcomes of initiatives that can complement quantitative results. The use of stories, however, is not widely recognized, and the methods and tools available to develop stories are limited. Therefore, we describe the methods used to collect, develop, and disseminate stories featuring comprehensive obesity prevention efforts that various state health departments are planning and implementing. We also discuss potential challenges and provide recommendations that public health practitioners may consider when developing similar stories.

## Introduction

Because of an increased emphasis on implementing policy, systems, and environmental (PSE) strategies to support healthful eating and active living, opportunities to share experiences from the field are growing among public health practitioners. Stories, or focused narratives that convey a sequence of events, can serve as a potential vehicle for communicating obesity prevention and control efforts at federal, state, and local levels to a broad audience, including researchers, policy makers, and other practitioners (1–5). Stories have been widely used by nonhealth sectors such as business, education, and social sciences to showcase programs and services, and they have been used increasingly by the public health sector to illustrate processes and outcomes that are not captured by quantitative methods (6).

The collection, development, and dissemination of stories from the field can support the advancement, assessment, and translation of public health initiatives. To advance public health efforts, stories can be shared with policy makers, funders, and local news media to bring increased visibility to an initiative, demonstrate the value of funded projects, document a return on investment or cost savings, and mobilize and garner needed resources (3,5–11). When disseminated to priority populations, stories can make people aware of an initiative and motivate and support PSE changes (2).

Stories also represent a tool for assessing and evaluating public health policies and practices. Stories can be developed throughout all stages of an initiative to communicate results and accomplishments to key stakeholders, particularly in the early phases of an initiative when outcomes cannot be fully determined from surveillance data or anticipated outcomes are not yet achieved (3,5,6). Distinct from quantitative epidemiologic studies or evaluation methods, stories rich in detail can capture results of collaboration with individuals and organizations; communicate successes, barriers, and lessons learned; and describe both intended and unintended consequences that span varying stages of the initiative, which are often challenging to convey with numbers only (1,3,6,11).

To bridge the translational gaps between practitioners, researchers, and policy makers, stories developed by practitioners can inform key stakeholders of public health policies and practices being implemented in the field (4,5,11–13). When communicated to other practitioners, stories can also translate critical information and guidance on how similar initiatives should be implemented. This information can include descriptions of where, when, and how an initiative was implemented; why it was successful; and key considerations for practitioners implementing similar efforts (6).

Despite the numerous benefits that stories can provide to individuals and organizations, practitioners do not often use this method of describing the processes or outcomes of a public health policy or practice. Stories may not be developed regularly because of the perceived subjectivity and susceptibility to bias and because of the traditional reliance on quantitative outcomes in public health (1,3,5,6,11,13). In public health organizations, the limited staff capacity, resources, and expertise to construct an effective story may also influence the adoption of stories. Similarly, Lewis et al have suggested that the methods and tools for practitioners or organizations to develop stories are infrequently disseminated (6). More specifically, as the implementation of PSE strategies are increasingly emphasized in chronic disease prevention, the limited methods and tools available for the development of such stories do not address the challenges associated with communicating more comprehensive results. Implementing a PSE change can be a lengthy and complex process, requiring numerous steps and engagement from multiple sectors (14). As a result, documenting the implementation of PSE strategies may not be as straightforward as stories featuring programmatic efforts describing the processes or outcomes related to individual program participants.

We describe the methods and tools used by the Division of Nutrition, Physical Activity, and Obesity (DNPAO) at the Centers for Disease Control and Prevention (CDC), in collaboration with state health departments funded through the State-Based Nutrition, Physical Activity, and Obesity (NPAO) Program, to collect, develop, and disseminate stories from the field as well as recommendations and challenges practitioners may consider when developing similar stories.

## Program Background

CDC’s DNPAO provides funding and support to state health departments through the State-Based NPAO Program. The primary focus of the state-based program is to implement PSE strategies that improve dietary quality, increase physical activity, and reduce obesity across multiple settings, including schools, worksites, health care settings, and other community venues (15). State health departments are encouraged to leverage resources and coordinate statewide efforts with multiple partners to address the following 6 principal target areas: 1) increase physical activity, 2) increase consumption of fruits and vegetables, 3) decrease consumption of sugar drinks, 4) reduce consumption of energy-dense foods, 5) increase breast-feeding initiation and duration, and 6) decrease television viewing (15).

To evaluate the overall state-based program, document activities, and understand success in PSE changes, state health departments are required to report progress annually through a web-based survey called the State Program Interim Reporting System (SPIRS), in which 1 component is the submission of a story from the field. SPIRS was designed to monitor activities and progress of state health departments and to assist CDC staff in providing states with improved technical assistance. Because of the interim nature of this reporting system, a popular online survey platform was used, which allowed for the secure transmission of data directly to CDC staff.

## Steps to Develop Stories

The steps reported by Lewis et al to develop a health promotion success story were tailored to collect, develop, and disseminate the DNPAO stories from the field (6). The adapted steps are detailed below and in the Box. Appendix A has an example of a DNPAO story from the field (also available at www.cdc.gov/obesity/stateprograms/statestories.html).

**Box. Comparison of Steps Used to Develop Stories**

Steps to Develop WISEWOMAN Success Stories (6)

1. Identify audience and purpose

2. Develop systematic process

3. Develop standardized form

4. Collect story ideas

5. Conduct interviews

6. Develop appealing format

7. Write and revise stories

8. Organize stories

9. Design and print publication

10. Disseminate publication

Steps to Develop DNPAO Stories From the Field

1. Identify audience and purpose

2. Collect story data

3. Review and select stories

4. Collect additional data

5. Develop and refine stories

6. Design story template

7. Disseminate stories

Abbreviations: WISEWOMAN, Well-Integrated Screening and Evaluation for Women Across the Nation; DNPAO, Division of Nutrition, Physical Activity, and Obesity of the Centers for Disease Control and Prevention.

**Identify audience and purpose.** The purpose of collecting DNPAO stories from the field was to develop stories that described efforts of state health departments and their role to support communities in planning and implementing PSE strategies related to healthful eating and active living. In comparison to traditional success stories, which only describe exemplary work and highlight achievements, these stories would represent states at various stages of development or maturity, including states that may have achieved full implementation of their PSE initiatives, states that may still be in early planning stages of an initiative, and states that may have had limited success. Overall, some of the goals of the stories would be to feature a diverse set of accomplishments, challenges, and lessons learned of state health departments and their partners; enhance horizontal consultation among state health departments; and increase knowledge of obesity prevention practices.

The primary audience for the DNPAO stories from the field was state health department staff involved in NPAO initiatives and practitioners in local health departments and partnering organizations implementing similar policies and practices. To increase visibility of state health department efforts and inform key stakeholders of the state-based program about practices in the field, secondary audiences of the stories were also identified, which included policy makers, researchers, CDC staff, and the general public.

**Collect story data.** In October 2010, twenty-five funded state health departments submitted a story through a web-based data collection form in SPIRS (Appendix B). Questions and in-depth directions were included in the data collection form to assist state health department staff, usually program managers, in the development of a story. Story data collected included background on the public health problem being addressed; a description of the target population; details on the process of planning or implementing the initiative, including partners involved, where and when the initiative was implemented, and resources associated with the initiative; a description of the key results or products of the initiative; a reflection on the facilitators and barriers to planning and implementation; and considerations for other organizations or communities implementing similar initiatives.

**Review and select stories.** Because only a limited number of stories could be developed from the data collected through SPIRS, criteria were applied for selecting the stories featured in the series. Two story project team members individually reviewed and ranked the 25 story submissions using the following criteria: focused on PSE approaches; addressed health equity; described collaboration with community-level partners; and demonstrated substantial reach, impact, replicability, and sustainability (Table). The story rankings were discussed and consideration was given to generating a collective set of stories that represented varied stages of development, DNPAO target areas, and settings. The 2 reviewers achieved consensus on the story rankings and 10 state stories were selected for development.

**Table:** Criteria Applied to Select DNPAO Stories From the Field

Category	Criteria
PSE approaches	Described state activities to create, maintain, or enhance PSE changes that will address obesity through various nutrition and physical activity strategies.
Health equity	Described state activities to address differences in incidence, prevalence, mortality, burden of diseases, and other adverse health conditions or outcomes that exist among specific-populations in a state.
Collaboration with community-level partners	Described collaborative efforts between the state health department and communities to support nutrition, physical activity, and obesity prevention efforts.
Potential reach	State activities that have the potential to affect a large proportion of the intended target population.
Potential impact	State activities that have the potential to have a meaningful effect for the target population.
Ability to replicate	State activities can be duplicated and similar effects can be achieved by similar entities.
Ability to sustain	State activities and outcomes can likely continue without intensive resources.

Abbreviations: DNPAO, Division of Nutrition, Physical Activity, and Obesity of the Centers for Disease Control and Prevention; PSE, policy, systems, and environmental.

**Collect additional data.** Following story review and selection, states were contacted by CDC staff to schedule telephone interviews with state health department staff and relevant community partners to further develop the story. Semistructured discussion guides (Appendix C) were developed for each state to fill in any missing gaps in the data collection form, provide additional details, and obtain quotes. The telephone interviews typically lasted 60 minutes and were audio recorded. A skilled interviewer facilitated the conversations and a note taker recorded detailed notes, including verbatim quotes. The interview participants were also asked to provide any available background materials, such as previous evaluation reports or web links on the initiative, that would further the development of the story.

**Develop and refine stories.** Data collected from each story submission and state interview were synthesized into a 1- to 2-page story. Stories were generally written using the following format: 1) a title, communicating the theme or focus of the story; 2) details on the policy or environmental change initiative, describing the key elements of the featured initiative and where appropriate, “how to” information that would be relevant to practitioners engaging in similar efforts; 3) short- and long-term results of the initiative, describing the main outcomes of the featured initiative, both intended and unintended, and where appropriate, information about new partnerships formed, organizational changes, and the potential public health effects; 4) lessons learned, summarizing the knowledge and experiences that facilitated or challenged the state efforts, which may be helpful to practitioners engaging in similar efforts; and 5) contact information.

Once the initial draft of the story was developed, it was shared with CDC staff and state health department representatives involved in the featured story for their review. The purpose of this review was to ensure that the story accurately represented the data collected from the story submission and telephone interview. The stories were revised and edited using any feedback received.

**Design story template.** Graphic designers developed a visually appealing template and selected appropriate photographs for each of the stories. Because it was anticipated that the stories would be posted on the CDC website, designers also ensured each story complied with regulations that require federal agencies to make information technology accessible to people with disabilities (16).

**Disseminate stories.** Stories were disseminated to state and local health departments, policy makers, researchers, CDC staff, and the general public through various e-mail listserves and CDC websites. State health departments featured in the series were encouraged to further disseminate the stories to their state- and community-level partners. The series and the methods by which the stories were developed were also shared at national conferences to create awareness of the methods and tools used and at trainings attended by various public health professionals to build capacity in story development.

## Limitations and Challenges

We encountered several limitations and challenges during the collection, development, and dissemination of the DNPAO stories from the field. First, the development of stories relied on recalled information for short- and long-term results, which potentially created recall bias. Second, the initial collection of story data needed to be supplemented with additional details and quotes. As a result, several rounds of revisions between the CDC writer, an editor, and contributors in the states were necessary. This revision process was often time-intensive, sometimes spanning several weeks to reach consensus on a final version of a story. Next, stories received through SPIRS varied in written quality. Although state health department representatives were most familiar with their initiatives, several did not demonstrate the expertise in developing a strong story from the field. The variability in the written quality of stories received may have also been due to the questions incorporated in the data collection form. The questions could potentially be revised to elicit responses that would support the development of more compelling and relevant stories. Finally, the stories did not comprehensively depict efforts from all 25 state health departments funded through the state-based program. However, in addition to capturing stories through SPIRS, DNPAO has encouraged states to develop their own state- and community-level stories independently of CDC to communicate their activities and outcomes. Effective story examples developed independently of CDC by state health departments include stories from Indiana (http://www.inhealthyweight.org/200.htm), Minnesota (http://www.health.state.mn.us/divs/cfh/connect/index.cfm?article=phstories.welcome), and North Carolina (http://publichealth.nc.gov/hnc2020/stories.htm).

## Recommendations

**Communicate to key stakeholders early. **Before story development, engage key stakeholders, such as story contributors, reviewers, and graphic designers, to gather input on the intended audience and purpose of the stories and to discuss the expected tasks and timelines associated with the story development process (3,6). Early discussions with key stakeholders can also guide future dissemination efforts.

**Collect story data systematically.** Determine a systematic method to solicit representative stories, which may include sending e-mail requests to potential story contributors, posting the request to an organization’s website or partnering organizations’ websites, or integrating the submission into ongoing evaluation or reporting activities.

Once a solicitation method has been selected, use a data collection form that has been vetted with potential story contributors. Consider incorporating focused questions that prompt responses that will support the development of appropriate stories. Supplementary questions that are tailored to a specific public health topic could also be included in the data collection form.

Interviews with key individuals involved in the initiative may either be used as a primary data source for story development or as a secondary data source to supplement information previously collected. For both scenarios, consider the most appropriate interviewee and interviewer for the discussion. While developing the DNPAO stories from the field, we encountered several state health departments that experienced staff turnover and directed us to other staff or community members who could describe the initiative similarly. Furthermore, a skilled interviewer comfortable with guiding a discussion, at times with multiple individuals, and asking probe questions should prepare a semistructured discussion guide and facilitate the conversation. Record the complete interview and use the audio recording as a reference when developing the story.

**Use experienced writers. **An understanding of the content areas addressed in the stories and strong writing skills are essential to developing appropriate stories for the intended audience (6). Consider designating staff members with backgrounds in creative writing or communications to develop the stories. For organizations lacking staff members with this related experience, explore ways to build staff capacity within the organization or partnering organizations to write quality stories, which could include writing workshops or conferences.

**Incorporate varied stories.** If developing multiple stories, consider the diversity of the stories featured across the larger series to ensure that the information included resonates with various audiences. For example, in addition to the criteria applied to select the stories presented in the DNPAO stories from the field, we sought to include stories that represented a diverse set of target areas, settings, geographic locations, and project stages (eg, variation in process- and outcome-focused stories). A diverse collection of stories can also be represented in additional ways, from featuring stories of organizations with contrasting financial resources or initiatives to working with different socioeconomic or ethnic populations.

**Understand the role of featured organizations and partners. **Individuals, organizations, and partners play various roles in supporting an initiative, and their diverse activities must be accurately recognized and represented in a story. Although several state health departments included in the DNPAO stories from the field supported policy changes related to healthy eating and active living, it was necessary to clarify their role in educating policy makers, assembling partners to prioritize efforts, and monitoring policy changes across a community or state.

**Dedicate sufficient time for review and graphic design. **Allocate ample time for story contributors and reviewers to revise and edit stories. Several state health departments featured in the DNPAO stories from the field required final approval from a communications coordinator or project director, which often postponed story development.

Organizing the final story format, in addition to selecting photographs for the story, can be time-intensive. Whether collaborating with a graphic designer on these tasks or not, dedicate adequate time for developing the story design early in the process, if possible.

**Explore innovative dissemination methods. **Stories can be disseminated by using novel methods. If resources allow, consider audio or video accompaniments to the stories, including podcasts or video vignettes. Explore opportunities to disseminate the stories using local media sources, social media outlets, and web-based story banks.

## Conclusion

Stories from the field have the potential to advance, assess, and translate promising DNPAO policies and practices. Stories can complement quantitative data by featuring accomplishments, demonstrating the value of various initiatives, describing lessons learned to help shape practices in the field, and engaging broader discussions of promising PSE approaches to support healthy eating and active living. Practitioners interested in documenting and sharing their efforts should gather and develop stories during the planning, development, and implementation stages of their initiative and collaborate with partners to innovatively disseminate the stories. Both practitioners and researchers should consider documenting, disseminating, and evaluating the methods and tools by which stories can be developed more efficiently.

## Appendix A. Sample Story From the Field

This file is available for download as an Adobe Acrobat Reader document.

## Appendix B. Data Collection Form

**Table:**

Question	Instructions
1. Please provide contact information for the story, including state, name, phone number, sand email address.	Provide the information requested. CDC staff may contact this person to follow-up on aspects of the story you provide. This person can be internal or external to the state health department.
2. What is the theme or focus for your story?	Before selecting a theme, think about the goal and audience of your story. What do you want the reader to do or know?
3. Please provide a title for your story.	Your title is one of the most important aspects of your story. Provide a title that captures the attention of your reader using action verbs and reflects the main theme of your story. Newspaper articles often use great bylines, so you may want to view a few for practice. Include the name of the program and try to avoid the use of acronyms.
4. What level of the socio-ecological framework does your story address?	Select all levels that apply to the story.
a. Individual	
b. Interpersonal	
c. Organizational	
d. Community	
e. Society	
5. What target area(s) does your story address?	Select the target area your story addresses, if applicable.
a. Increase physical activity	
b. Increase consumption of fruits and vegetables	
c. Decrease the consumption of sugar-sweetened beverages	
d. Reduce the consumption of high energy dense foods	
e. Increase breastfeeding initiation and duration	
f. Decrease television viewing	
6. What need did your efforts address?	Define the problem and make the case for why you approached this issue. Be sure to identify your target population. Be sure to provide a strong lead sentence.
7. Please explain the actions you took. Be sure to include all parties involved and any costs or other resources associated with your efforts. Please provide sufficient detail in case others would like to replicate your actions.	Provide step-by-step details about your process, including the specific strategies that were involved, who was involved, where and when your initiative was implemented, and how long it took to plan or implement your efforts.
8. Please describe the results of your efforts, both intended and unintended. Where appropriate, please include information about.	Summarize the key results or products of your efforts, if applicable. Be sure to include intended and unintended results. Unintended results can include: 1) a positive unexpected benefit; 2) a negative or perverse effect that is contrary to what was originally intended; or 3) a potential source of problems. If available, include data or memorable facts that support your story.
a. New partnerships formed	
b. New organizational processes	
c. How your approach led to a more effective program	
d. The potential public health impact of your efforts	
9. If possible, please include a specific quote from program staff or partners that would support your story.	Stories are more interesting if you provide quotes. Please include quotes that illustrate the ideas you have presented and evoke emotion in your readers. The quote does not have to come from the primary contact for the story and can be internal or external to the state health department.
10. Please describe the 3 key elements that facilitated your efforts. Examples of potential facilitating elements include:	Please describe what facilitators helped you during the planning and implementation of your efforts. Be sure to describe expected and unexpected facilitators.
a. Specific resources that facilitated your efforts	
b. Support from particular stakeholders	
c. Partnerships with new or existing partners	
11. Please describe the challenges or barriers you faced in your efforts.	Please describe what barriers inhibited you during the planning and implementation of your efforts. Be sure to describe expected and unexpected barriers. If you did not encounter any barriers, to what do you attribute your experience?
12. Please describe how your organization was able to overcome the challenges or barriers you described above. If you were not able to, what could help our organization to overcome these challenges?	Provide the information requested.
13. What tips do you have for using or adapting this approach in another organization, community, or state?	Describe “points for consideration” to which others implementing similar approaches should be mindful.
14. What would your organization do differently to enhance your planning and implementation processes related to this effort?	Reflect on your experiences and lessons learned. What did you not know about the initiative that you know now? How was this information helpful and how will it impact your future efforts?

## Appendix C. Sample Discussion Guide

This file is available for download as a Microsoft Word document.
