# Supplementary material for: Developing Stories From the Field to Highlight Policy, Systems, and Environmental Approaches in Obesity Prevention
Source: Prev Chronic Dis. 2013 Feb 14;10:E23. doi: 10.5888/pcd10.120141 (PMC3604804; doi:10.5888/pcd10.120141)
Supplement: Supplementary file 1 [file 12_0141appA.pdf]

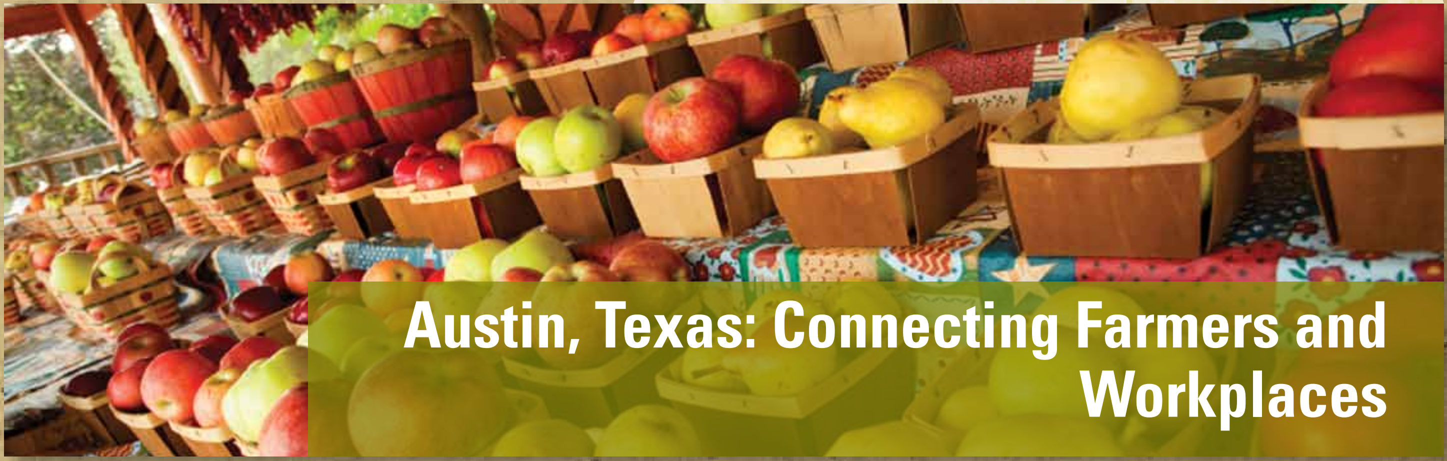

Austin, Texas: Connecting Farmers and Workplaces

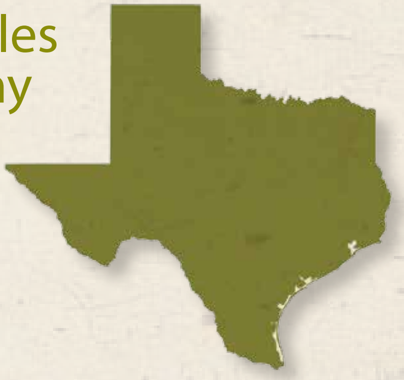

Incorporating more fresh fruits and vegetables into our daily fare is a recurring item on many lists of New Year's resolutions. In practice, though, it's not always as easy as it sounds. To make access to fresh fruits and vegetables more convenient—and thus more likely—a partnership between Austin's nonprofit Sustainable Food Center (SFC) and the Texas Department of State Health Services (DSHS) brings the farm directly to the workplace.

The Farm to Work program started in Austin, Texas in 2007, when DSHS staff approached SFC for help launching a program as part of a workplace wellness initiative within state agencies. SFC—a local nonprofit whose mission is to strengthen the local food system and to improve access to nutritious, affordable food—was a natural choice. Its staff already had experience connecting farms to large organizations; such as, universities and hospitals, so they had many ideas for DSHS.

> **Since the initial launch in 2007**, nearly **30 work sites in Central Texas** now participate in **Farm to Work**, including the state Comptroller's Office and the Departments of Agriculture and Education. Few work sites have withdrawn and **work sites around the state** and in other parts of the United States have **used the toolkit to start their own version** of Farm to Work.

A Hybrid Model, Fueled by a Web Site

SFC and DSHS considered several options, including starting a farmers' market on site, but ultimately decided that a hybrid Community Supported Agriculture (CSA) subscription model would work best for large state agencies. In most CSA models, people sign up for a share of a local farm's produce and receive a basket of seasonal fruits and vegetables each week, paid in advance at the beginning of a growing season. In the SFC/DSHS version, employees can still sign up for a share of local farm's harvest, but instead do so on a week-to-week basis, via a secure Web site that sets up the whole ordering and payment transaction between individual employees and local farmers.

The hybrid CSA model was a good choice for several reasons. First, it allowed employees to try the delivery system without making a long or expensive commitment. Each basket costs $20, 13% less than a comparable bag of produce at a local grocery store, and offers a variety of different seasonal items, listed ahead of time on the ordering Web site. Second, because the transaction is directly between the farmer and the individual employee, this model removed the state agency from foreseeable legal obstacles to hosting the program.

The link between farmer and employee may be facilitated by the SFC and DSHS partnership, but it relies on a highly functional Web site that makes ordering and payments easy. In addition to making the online payment process smooth and hassle-free, the Web site doubles as a promotional tool and even links to recipes, including a collection of healthy recipes from the Centers for Disease Control and Prevention's Web site.

Essentially, SFC and DSHS play a matchmaking role between local Austin farmers and potential customers already gathered in one location, a workplace. SFC identifies local farmers and assesses their interest and production capacity, while DSHS determines the level of interest among employees and work sites within the farmers' geographic radius. Once the initial matchmaking is underway, however, a representative from each of the participating work sites takes over site-specific tasks, heading up weekly outreach, special promotions, and meetings with the farmers to manage the distribution each week. This involvement from a work site representative encourages ownership and sustainability of the program.

A Toolkit Guides Expansion

As word of the program spread, DSHS and SFC were approached by other agencies and organizations whose staff was eager to start similar programs. The partners were pleased by these requests, because they had always intended to start a model that could be replicated, rather than a stand-alone program. To help others interested in replicating Austin's Farm to Work program, DSHS and SFS captured what they had learned so far in a comprehensive Farm to Work Toolkit, which continues to be updated as they learn more about effective implementation.

The toolkit reviews the main program components and walks prospective program coordinators through each step, from legal concerns to assessments (including online surveys of both farmers and employees), timelines, checklists, budget estimates, the logistics of distributing the produce, staffing the program, and evaluating it. To make replication even simpler, the toolkit and a companion resource guide include sample legal forms, marketing posters, and screen shots from the ordering Web site.

The marketing materials and Web site deliberately play down the role of DSHS and SFC so that each participating workplace can claim—and brand—its Farm to Work initiative as its very own.

Since the initial launch in 2007, nearly 30 work sites in Central Texas now participate in Farm to Work, including the state Comptroller's Office and the Departments of Agriculture and Education. Few work sites have withdrawn and work sites around the state and in other parts of the United States have used the toolkit to start their own version of Farm to Work.

---

[1] See ***www.dshs.state.tx.us/obesity/nutritionfarmtowork.shtm*** for a downloadable copy of the Toolkit and other materials.

Lessons Learned

The toolkit offers a many tips and strategies, but a few are worth highlighting. First, the role of an onsite, local coordinator is crucial. Without this internal champion, the program is unlikely to launch, let alone last. The time commitment initially is fairly extensive, but tapers quickly once the system is in place and running smoothly. The same is true of the initial tasks of building and testing the Web site and navigating legal issues: once they are in place, they require little time from staff. Another crucial early task is to obtain high-level buy-in from the employer's leadership. This helps with addressing legal obstacles as well as marketing and outreach.

So far, employees have been steady and enthusiastic participants. For them, the benefits include more fruits and vegetables on dinner tables and in lunchboxes, and social support to do so. The benefits accrue to employers, as well. By helping to make the healthy choice the easy choice, employers are providing wellness to their employees.

What's next? Slowly but steadily, Farm to Work plans to expand across Texas, matching more farmers and workplaces to each other. As one of the partners says only half-jokingly... First, Farm to Work—then, Farm to World! Between the new manager and the new look, the farmers' market made a profit for the first time.

For more information, please contact:

Maribel Garcia Valls
Texas Department of State Health Services
Email: maribel.valls@dshs.state.tx.us

Tamara Lewis
Texas Department of State Health Services
Email: tamara.lewis@dshs.state.tx.us

 This initiative was supported by CDC cooperative agreement CDC-DP08-805. Developed September 2011
