# Supplementary material for: Developing Stories From the Field to Highlight Policy, Systems, and Environmental Approaches in Obesity Prevention
Source: Prev Chronic Dis. 2013 Feb 14;10:E23. doi: 10.5888/pcd10.120141 (PMC3604804; doi:10.5888/pcd10.120141)
Supplement: Supplementary file 2 [file 12_0141appC.docx]

**Appendix C. Sample Discussion Guide**

Introduction Script:

*Hello ________:*

*We are working to gather stories from state health departments that highlight achievements and lessons learned from state program efforts.*

*The information gathered today, as well as the information provided in your original story submission will be incorporated into a story about your agency’s work in nutrition, physical activity, and obesity prevention. We would like to record this conversation to ensure we capture all pertinent information. Do you have any objection to me recording our conversation? Thanks.*

*Do you have any questions for me before we start the interview? Then let’s get started.*

Interview Questions:

Background

1. Please tell us a little about how the Farm to Work program and the Texas Farm to Work Toolkit.
2. Why did the Texas Department of State Health Services decide to expand the Farm to Work program?

Planning and Implementation

1. Tell us about the partners that were involved in the expansion of the program.
   1. What were the roles of each of these partners during the expansion of the program?
2. What steps were involved in *preparing* for the expansion of the Farm to Work program?
   1. How were worksites selected to participate?
   2. How were farmers selected to participate?
3. What steps were involved with *implementing* the expansion of the Farm to Work program?
4. What type of training, technical assistance, or support did the Texas Department of State Health Services provide to worksites and farmers during the expansion?
5. Tell us how worksites outside of these organized expansion efforts are utilizing the Texas Farm to Work toolkit or adapting the Farm to Work program.
   1. Do you have an idea of how many worksites are implementing similar initiatives?
   2. What type of training, technical assistance, or support does Texas DSHS provide to similar initiatives?

Results

1. What was the immediate response from worksites and farmers participating in the program?
2. Do you have any information on the number of employees reached or healthy foods sold as a result of these initiatives?

Facilitators and Barriers

1. What were some of the major barriers you encountered while expanding the Texas Farm to Work program?
   1. How did you overcome those challenges?
2. What part of the planning and implementation was most time intensive?
3. For other state health departments considering a similar initiative, what would you recommend?

Next Steps

1. What are the next steps for the program?
2. Is there anything else you would like to share with us about the program?

Ending Script

*I would like to thank you for your time and insights. Once the story draft has been developed, we would like to share it with you and ask for your review and revisions. We anticipate sending that to you around [Insert date] and would ask that you get comments back to us in about a week. Will you be available at that time; will that timeframe work for you? Great. [Confirm appropriate contact and contact information]. Please also feel free to reach out to me if you have any additional ideas or if we can answer any questions.*
